# Sperm competition experiments reveal low prezygotic postmating isolation between parasitic and nonparasitic lamprey ecotypes

**DOI:** 10.1002/ece3.9970

**Published:** 2023-04-02

**Authors:** Nolwenn Decanter, Romane Normand, Ahmed Souissi, Catherine Labbé, Eric Edeline, Guillaume Evanno

**Affiliations:** ^1^ DECOD (Ecosystem Dynamics and Sustainability), INRAE, Institut Agro, IFREMER Rennes France; ^2^ INRAE, UMR1037 LPGP, Fish Physiology and Genomics Campus de Beaulieu 35000 Rennes France

**Keywords:** cryptic female choice, ecotype, postmating prezygotic barrier, reproductive isolation, speciation, sperm traits

## Abstract

The role of postmating sexual selection as a potential reproductive barrier in speciation is not well understood. Here, we studied the effects of sperm competition and cryptic female choice as putative postmating barriers in two lamprey ecotypes with a partial reproductive isolation. The European river lamprey *Lampetra fluviatilis* is anadromous and parasitic of other fish species, whereas the brook lamprey *Lampetra planeri* is freshwater resident and nonparasitic. We measured sperm traits in both ecotypes and designed sperm competition experiments to test the occurrence of cryptic female choice. We also performed sperm competition experiments either at equal semen volume or equal sperm number to investigate the role of sperm velocity on fertilization success. We observed distinct sperm traits between ecotypes with a higher sperm concentration and a lower sperm velocity for *L. planeri* compared with *L. fluviatilis*. The outcomes of sperm competition reflected these differences in sperm traits, and there was no evidence for cryptic female choice irrespective of female ecotype. At equal semen volume, *L. planeri* males had a higher fertilization success than *L. fluviatilis* and vice versa at equal sperm number. Our results demonstrate that different sperm traits between ecotypes can influence the male reproductive success and thus gene flow between *L. planeri* and *L. fluviatilis*. However, postmating prezygotic barriers are absent and thus cannot explain the partial reproductive isolation between ecotypes.

## Graphical abstract

This study analyzes the effects of sperm competition and cryptic female choice as putative postcopulatory barriers in two lamprey ecotypes with a partial reproductive isolation. We observed different sperm traits between ecotypes, which influenced the outcome of sperm competition experiments, but we found no evidence for cryptic female choice.

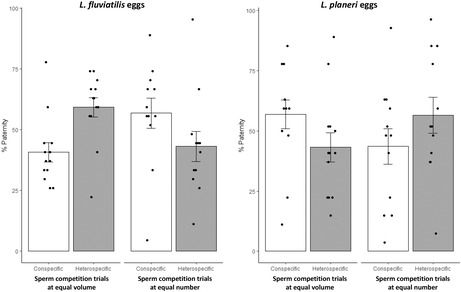

## 1 INTRODUCTION

Speciation can be defined as the evolution of reproductive isolation within an ancestral species, resulting in two or more descendant species (Rabosky, 2016). Natural and sexual selection can both influence reproductive isolation, i.e., the evolution of traits that reduce gene flow between populations (Kirkpatrick & Ravigné, 2002). The role of natural selection in speciation has been widely studied since Darwin's pioneering work on this topic (Darwin, 1859), but there is a lack of consensus about the role of sexual selection in this process (Safran et al., 2013). Sexual selection can drive speciation through precopulatory mechanisms like male–male competition and female choice but also postcopulatory processes that include sperm competition and cryptic female choice (Andersson, 1994; Qvarnström et al., 2012; Tinghitella et al., 2018, Rundle & Rowe, 2018). Sperm competition occurs when sperm from two or more males are in direct competition to fertilize ova (Edward et al., 2015), and cryptic female choice occurs when females can bias sperm utilization and thus paternity (Firman et al., 2017). Cryptic female choice can promote speciation by disfavoring heterospecific sperm fertilization through a better siring success of conspecific males compared with heterospecific males (Yeates et al., 2013). Recently, Simmons (2018) suggested that extremely rapid evolutionary divergence can be driven by sperm competition due to cycles of antagonistic coevolution between males and females. However, the role of sperm competition in speciation has received very little attention compared with precopulatory male competition (examples reviewed in Qvarnström et al., 2012 and Tinghitella et al., 2018).

Lampreys are jawless fishes that represent one of the oldest living groups of vertebrates and have become model species for studying speciation (Docker, 2015). They reproduce in freshwater and are external fertilizers. A number of lamprey species are known as “paired species” that are closely related species with contrasting migration and feeding strategies (Lasne et al., 2010; Rougemont et al., 2015). In most pairs, one form is anadromous and parasitizes other fish species after metamorphosis whereas the other form is freshwater‐resident and nonparasitic (Zanandrea, 1959). During reproduction, both forms can sometimes be observed on the same spawning grounds and hybridize, hence depending on the level of reproductive isolation, these forms can be considered as ecotypes or species (Docker, 2015).

The European river lamprey *Lampetra fluviatilis* and brook lamprey *L. planeri* are the most studied paired lamprey species (Salewski, 2003), but their level of speciation is still debated (Lasne et al., 2010; Mateus et al., 2013; Rougemont et al., 2015). They were recently considered as partially reproductively isolated ecotypes, hence for this study we will refer to them as “ecotypes” (Rougemont et al., 2017, 2021). *L. fluviatilis* is parasitic‐anadromous and is mainly distinguished from the nonparasitic freshwater‐resident *L. planeri* by its larger body size at the adult stage. A high frequency of communal spawning (i.e., spawning of several males and females on the same nest) involving both ecotypes was reported in a French coastal river (Lasne et al., 2010). In particular, *L. planeri* males were observed while trying to mate with *L. fluviatilis* females. Rougemont et al. (2015) observed in a semi‐natural setting that *L. planeri* males can fertilize *L. fluviatilis* eggs and there was no difference in hatching rate between within‐ecotype and between‐ecotype crosses in both cross directions (see also Hume et al., 2013a; Staponkus & Kesminas, 2014). In addition, Hume et al. (2013b) reported that *L. planeri* males may adopt a sneaking tactic to fertilize *L. fluviatilis* eggs and *L. fluviatilis* males could also mate and adopt a sneaking tactic with females of a smaller resident‐parasitic ecotype (see also Hume et al., 2018). This widespread tactic in fish consists in exploiting the reproductive investment of large dominant males that secure mates and/or defend breeding territories whereas sneaker males are usually smaller than dominant males, do not defend any territories or mates, and will sneak fertilization during mating of females with dominant males (Taborsky, 1997). Sneaker males tend to have larger testes, and produce more and faster sperm than others (Flannery et al., 2013; Lehnert et al., 2017; Miller et al., 2019; Poli et al., 2018; Rasotto & Mazzoldi, 2002; Scaggiante et al., 1999). In lampreys, the gonadosomatic index (GSI) appears to be higher in males of nonparasitic species (e.g., 11.9% for *L. planeri*, Maitland et al., 1994) compared with parasitic species (e.g., 4.64% in *L. fluviatilis*, Abou‐Seedo & Potter, 1979; Docker, 2019), but nothing is known on sperm traits and sperm competition in these taxa.

Here we assessed the extent of sperm competition and cryptic female choice in *L. fluviatilis* and *L. planeri* using in vitro fertilization experiments. First, we evaluated the sperm competitiveness of *L. fluviatilis* and *L. planeri* by comparing their sperm velocity and concentration. Second, we performed sperm competition experiments with gametes of both ecotypes to test the occurrence of cryptic female choice. In fish with external fertilization, the fertilization success is strongly linked to sperm traits like sperm velocity and sperm number (Gage et al., 2002, 2004; Lahnsteiner et al., 1998). Consequently, two types of competitive fertilizations were performed with a paired‐male experimental design on eggs of both ecotypes: (i) at equal semen volume and (ii) at equal sperm number. Our aim was to test for an effect of sperm velocity when sperm number was kept constant (Gage et al., 2002, 2004). In addition, noncompetitive fertilizations were performed as controls to check that the fertilization and hatching rates were similar for within‐ and between‐ecotype crosses. As *L. planeri* males have a higher GSI and may adopt a sneaking tactic, we hypothesized that they might produce ejaculates with a higher sperm number and velocity than *L. fluviatilis* males. In both types of sperm competition experiments and the absence of cryptic female choice, we hypothesized that *L. planeri* males should sire more offspring due to putative better semen quality. Alternatively, if cryptic female choice does occur, we predict that for eggs of one ecotype, sperm from the same ecotype should have a higher success in all experiments.

## 2 MATERIALS AND METHODS

### 2.1 Sampling

We collected adult lamprey by electrofishing in April 2021 in the Oir River, France (48°37′39.7″N 1°16′26.5″W). In total, 54 *L. fluviatilis* (34 females and 20 males) and 87 *L. planeri* (51 females and 36 males) were caught. The lampreys were then kept in the INRAE research station of Cerisel (Ducey–France). They were anesthetized using 0.4 mL of benzocaine (0.02 mol L^−1^) for 1 L of water. Individual fin clips were sampled and preserved in 99.8% ethanol. Each lamprey was labeled with individually visible implant elastomer (VIE) marks on the dorsal fin (Evans, 2017). Lampreys were kept in four 250‐L tanks with males and females being separated. The water temperature was adjusted to 12°C in each tank. The sperm quality analyses were conducted 5 to 30 min after stripping.

### 2.2 Detection of hybrids

We genotyped all individuals with a diagnostic SNP developed by Souissi et al. (2022) in order to detect hybrids since there is a high level of genetic admixture between *L. fluviatilis* and *L. planeri* in the Oir population (Rougemont et al., 2015). *L. fluviatilis* and *L. planeri* individuals are homozygous at this marker called diagLpf (genotypes *ff* or *pp*, respectively) whereas hybrids are heterozygous (*pf*). We kept only homozygous individuals for competitive fertilization experiments in order to determine the paternity of offspring with the same marker. We used the kit NucleoSpin® 96 Tissue (Macherey‐Nagel, Düren, Germany) to extract DNA from fin clips according to the manufacturer's instructions. Then, we genotyped all individuals at the diagLpf locus with quantitative polymerase chain reactions (qPCR) by using the protocol of Souissi et al. (2022). The PCR products were run on a CFX96 Touch Deep Well Real‐Time PCR System (Bio‐Rad) and analyzed with CFX Maestro™ Software (version 4.1.2433.1219). We analyzed sperm traits for 26 males homozygous at the diagLpf locus: 13 *L. fluviatilis* and 13 *L. planeri*.

### 2.3 Sperm velocity measurement

Sperm velocity assessments were realized thanks to videos processed using a Computer‐assisted Sperm Analysis system called OpenCASA (available at https://github.com/calquezar/OpenCASA; Alquézar‐Baeta et al., 2019). OpenCASA is a plugin to the open‐source software Fiji (free software, https://imagej.net/software/fiji/downloads) that proved to be efficient to evaluate sperm parameters in fishes (Sanches et al., 2013).

As advised by Cosson (2019), a dark field optical microscopy with a high magnification (400×) objective lens was used to record the videos. To prevent sperm from sticking to the microscope blade, a 0.5% solution of bovine serum albumin (BSA) in distilled water was used. For every male, each 1 μL subsample of sperm was activated with the BSA solution at room temperature. The temperature in the room was kept constant and the sperm velocity measurements of males of the same pair were done sequentially 5 to 30 min after stripping. Following activation, the subsample was placed under a blade mounted on slat and immediately placed under the microscope. Recordings started approximately 17 seconds after movement activation (mean ± SE: 17.48 ± 2.2). The duration of lamprey sperm motility is high in comparison with many other freshwater fishes. For instance, the spermatozoa of the rainbow trout are motile for about 30 s whereas those of lampreys are motile for 5 min after activation in *Lampetra japonica* (Kobayashi, 1993), from 4 min to more than 10 min in the sea lamprey (Ciereszko et al., 2002) and at least 5 min in *L. planeri* and *L. fluviatilis* (personal observation). As a result, the average time we used to start the records was very short compared with the total duration of sperm motility. Sperm movements were registered with a Nikon D7500 camera adjusted on a Leica Leitz DMRB microscope (GMBH Germany 541,006). The recording speed was 60 frames per second. Videos were collected using Microsoft Photos (free software, https://www.microsoft.com/enus/p/microsoftphotos/9wzdncrfjbh4?activetab=pivot:overviewtab) and cut just after microscope focusing to keep 3 s for analyses and exported in mp4 format. In order to have an uncompressed avi format for OpenCASA, videos were formatted in the open‐source software PuTTY using the command “ffmpeg –i Video_name.mp4 –f avi –vcodec mpeg convertedFile.avi.” The videos were then opened in Fiji and analyzed using the OpenCASA plugin according to the procedures described by Alquézar‐Baeta et al. (2019). The settings used are available in Table S1.

The sperm parameters calculated by OpenCASA were the *curvilinear velocity* (VCL, i.e., the time‐averaged velocity of a sperm head along its actual curvilinear path) and the *average‐path velocity* (VAP, i.e., the time‐averaged velocity of a sperm head along its average path), all expressed in μm s^−1^ (Gallego et al., 2017). Sperm parameters were measured on three subsamples analyzed sequentially and originating from one stripping for each male. The average value over subsamples was used for statistical analyses. For each subsample, the average number of sperm cells tracked was 116.46 ± 7.8 (mean ± SE).

### 2.4 Sperm number quantification

Sperm number was determined using a Thoma cell counting chamber on three subsamples for each male. Each one μL subsample was diluted with a Ringer solution that consisted of 137 mM NaCl, 2.9 mM KCl, 2.1 mM CaCl_2_, and 2 mM Hepes (Kobayashi & Yamamoto, 1994). This solution allows the sperm to stay inactivated. Then, the sample was loaded in the Thoma cell counting chamber before the cover glass was put in position. As advised by Christensen et al. (2005), it was ensured that Newton rings could be observed between the pillars and the cover glass to make sure that the counting chamber was correctly mounted. After loading, the chamber was left in a wet environment for 4 min to allow the sperm to settle. The chamber was then placed under the Leica Leitz DMRB microscope (GMBH Germany 541,006) using 100× magnification and phase contrast. A picture was taken with a Nikon D7500 camera and counts were performed by using the software Fiji. The total number of sperm counted in the counting area was divided by the volume and multiplied by the dilution factor to estimate the sperm number in the original semen sample (Christensen et al., 2005). The average value over the three subsamples was used for statistical analyses.

### 2.5 In vitro fertilization experiments

Mature individuals were anesthetized using benzocaine and hand‐stripped to obtain gametes. The stripping and fertilization were performed on several days because the lampreys were not all mature at the same time (Table S2). Males and females were all stripped only once on a single day except one female who was stripped twice on two consecutive days (Table S2). For each in vitro fertilization, 6 μL of semen was exposed to a batch of eggs from one female in a Petri dish (mean ± SE = 77 ± 32 eggs). Then, the Petri dish was half‐filled with reconstituted water (OECD, 2008).

To compare the fertilization rate of *L. fluviatilis* and *L. planeri* eggs in within‐ecotype crosses and between‐ecotype crosses, noncompetitive in vitro fertilizations were realized (same approach as Yeates et al., 2013). For each female of both ecotypes, two egg batches were created and then fertilized using sperm from either *L. fluviatilis* or *L. planeri*. Consequently, 26 within‐ecotype and 26 between‐ecotype crosses were realized (Table S2).

To measure the fertilization success of males in competition, the experimental design was inspired from Yeates et al. (2013) and Gage et al. (2004). First, sperm competitions at equal semen volume consisted in exposing eggs to a homogenized mix of 3 μL *L. fluviatilis* and 3 μL *L. planeri* semen. Second, sperm competitions at equal sperm numbers consisted in exposing eggs to a different amount of semen from each male in order to have the same sperm number for both males (total semen volume = 6 μL). This was possible thanks to sperm number estimated with Thoma cell chambers.

Our aim was to produce 13 blocks with 13 male pairs and 13 female pairs, but due to a lack of mature females on the same day, we used some females for several blocks (Table S2). In total, 26 males (i.e., 13 male pairs) and 9 females (3 *L. fluviatilis* and 6 *L. planeri*) were used for in vitro fertilization (Table S2). However, the in vitro fertilization did not work for one male pair at equal sperm number (the eggs were not fertilized), hence only 12 male pairs were used for analyses at equal sperm number.

### 2.6 Eggs monitoring

A picture of each Petri dish was taken to evaluate the fertilization rate after sperm activation since fertilized eggs can be visually detected as they develop a perivitelline space whereas unfertilized ova do not (Rougemont et al., 2015). The Petri dishes were then placed in the dark into a climate chamber at 11.5°C ± 0.14°C (mean ± SE). Every 2 days, dead eggs were counted and removed in order to measure embryo survival.

We monitored embryo viability as hybrid embryos may suffer differential mortality and thus bias the results of sperm competition trials (Yeates et al., 2013). After hatching, at 27 days postfertilization, the number of alive embryos was counted. Embryo viability was then measured as the number of alive embryos divided by the number of eggs that were initially fertilized.

### 2.7 Paternity assignment

To identify the sires (i.e., *L. fluviatilis* or *L. planeri*) in each competitive fertilization trial, we genotyped 27 larvae from each trial at the diagLpf locus, except in one cross at equal sperm number for which only 22 larvae survived (total number of larvae genotyped = 1342). Larvae were collected 27 days postfertilization and DNA extractions and genotyping were performed as previously described. The paternity of each larva was determined according to one of three possible genotypes: *ff*, *pp*, or *pf*.

### 2.8 Data analysis

All statistical analyses were performed with the R software v. 4.0.3 and/or JAGS 4.3.0. Mann–Whitney tests were used to compare sperm numbers between *L. fluviatilis* (LF) and *L. planeri* (LP) due to normality deviation in residuals from parametric tests with those data. For the comparisons of velocity parameters (VCL and VAP), *t*‐tests were performed. Considering that *L. planeri* males have a GSI of 11.9% (Maitland et al., 1994), we estimated the total amount of sperm produced per male by multiplying this GSI value by the individual sperm number, assuming that 1 mL of sperm weighs 1 g. We did the same for *L. fluviatilis* males considering a GSI of 4.64% (Abou‐Seedo & Potter, 1979). We finally compared the total amount of sperm of *L. fluviatilis* and *L. planeri* males using a Mann–Whitney test.

Fertilization success of eggs exposed to sperm of both ecotypes was analyzed using GLMMs in the lme4 package or the MASS package when there was overdispersion. For *L. planeri* eggs, a GLMM with a binomial distribution and logit‐link function was used. For *L. fluviatilis* eggs, a GLMM with a quasibinomial family and logit‐link function was used because of overdispersion. The models included the male ecotype (LP or LF) as a fixed variable and female identity as a random effect. The significance of the fixed variable was determined using a likelihood‐ratio chi‐square test.

To analyze embryo viability, a GLMM was performed with a quasibinomial family and logit‐link function because of overdispersion (for both *L. fluviatilis* eggs and *L. planeri* eggs). The model included the cross‐type (within‐ecotype or between‐ecotype) as a fixed effect and female identity as a random effect. The significance of the cross‐type effect was determined using a likelihood‐ratio chi‐square test.

To test the effect of the sperm speed, female ecotype, and type of experiment (equal semen volume or number) on the siring success in between‐ecotypes crosses, we fitted a Bayesian GLMM with a binomial distribution and a logit‐link function. We used this flexible approach to easily estimate each model parameter and the uncertainty linked to each parameter. We used the following model:

$$C_{i}\;\mathrm{Bin}\left(p_{i},N_{i}\right)$$

$$\begin{array}{cc} \text{logit}\left(p_{i}\right)= & \propto_{0}+\propto_{1\left[{\text{female}}_{\mathrm{sp}}\left[i\right]\right]}+\propto_{2\left[\text{experiment}\left[i\right]\right]}+\propto_{3}*v{s_{\mathrm{LP}}}_{\left[i\right]}+\propto_{4}*v{s_{\mathrm{LF}}}_{\left[i\right]}\\ & +\propto_{5\left[\text{experiment}\left[i\right]\right]}*v{s_{\mathrm{LP}}}_{\left[i\right]}+\propto_{6\left[\text{experiment}\left[i\right]\right]}*v{s_{\mathrm{LF}}}_{\left[i\right]}+\beta_{1\left[\text{femal}{e_{\mathrm{id}}}_{\left[i\right]}\right]}\\ & +\beta_{2\left[{\text{male}}_{\text{pair}}\left[i\right]\right]}, \end{array}$$

where $C_{i}$ is the observed number of larvae sired by the LP male, Bin is a binomial distribution, $p_{i}$ is the probability of the male LP to be the sire, $N_{i}$ is the total number of larvae, $\propto_{0}$ is the fixed effect intercept; $\propto_{k^{\prime s}}$, $k\in \left\{1,\ldots ,6\right\}$ are the fixed effects associated with the female ecotype (LP or LF), the experiment type (equal volume or equal number), the LP sperm speed, the LF sperm speed, the interaction between the LP sperm speed and the experiment type, and the interaction between the LF sperm speed and the type of experiment, respectively. Sperm speeds were scaled to zero mean and unity standard deviation to avoid spurious correlations between intercept and slope. $\beta_{1}$ is the random effect associated with the female identity, and $\beta_{2}$ is the random effect associated with the male pair. For fixed effects the priors followed a t distribution while random effects followed a Gaussian distribution:

$$\propto_{i}\;\mathrm{dt}\left(\frac{1}{2.5^{2}}\right)i=0,\ldots ,6$$

$$\beta_{i}\;d\mathrm{norm}\left(0,\tau \right)$$

We estimated the parameters using JAGS v4.3.0 (Plummer, 2003) implemented in R v3.6.2 through the package jagsUI that relies on the packages rjags and coda. We ran four independent MCMC chains of 200,000 iterations each with a burn‐in of 100,000, and thinned chains at a period of 10 iterations. The convergence of the MCMC chains was assessed using the trace plots (Figure S2), Gelman‐Rubin $\hat{R}$ statistic (Gelman & Rubin, 1992) and a Bayesian *p*‐value (Gelman et al., 1996). The estimates of the random effects are provided in Table S3. Briefly, we computed residuals for the actual data and for synthetic data simulated from estimated model parameters (i.e., residuals from fitting the model to “ideal” data). The Bayesian *p*‐value is the proportion of simulations in which ideal residuals are larger than true residuals. If the model fits the data well, the Bayesian *p*‐value is close to .5. Bayesian *p*‐value for our model was .5237.

We tested the significance of effects from posterior parameter distributions with an MCMC *p*‐value obtained in a test equivalent to a two‐tailed *t*‐test. Specifically, the MCMC *p*‐value was twice the proportion of the posterior for which the sign was opposite to that of the mean posterior value. However, the MCMC *p*‐value for the effect of sperm speed in the equal sperm number trials was computed simply as the proportion of positive posterior values given the hypothesis that the higher sperm speed of LF males should confer them a higher siring success (i.e., equivalent to a one‐tailed test, see results).

We also tested whether both ecotypes had an overall different siring success, in each type of sperm competition experiment. At each MCMC iteration, we computed Δ as the difference between 0.5, corresponding to random paternity, and posterior probability for LP paternity of progeny from LP and LF mothers in both experiments (i.e., four probabilities). We then computed two‐tailed MCMC *p*‐values for Δ as described above.

## 3 RESULTS

**FIGURE 1:**
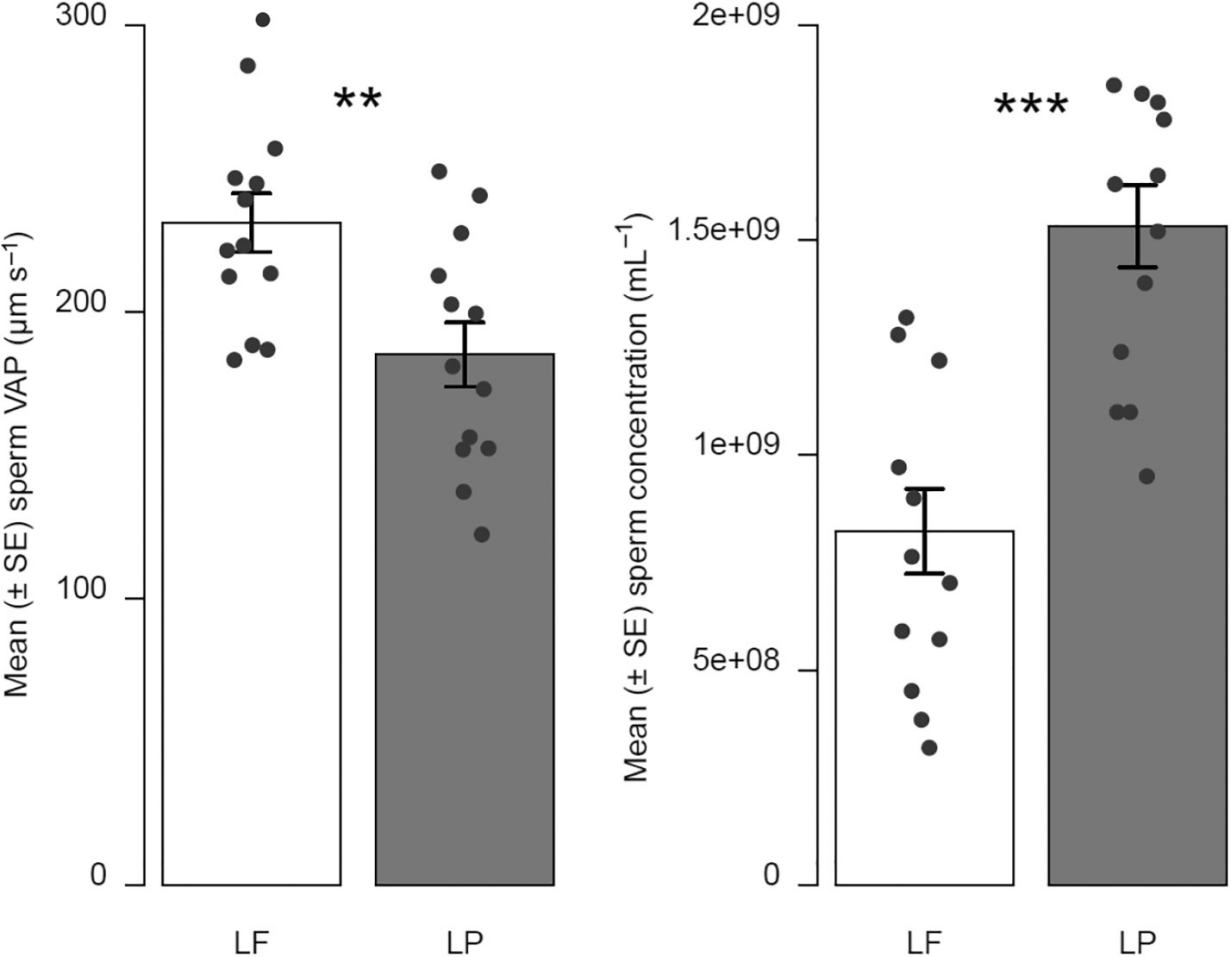
Comparison (mean ± SE) of sperm average‐path velocity (VAP) and sperm concentration between *Lampetra fluviatilis* (LF) and *Lampetra planeri* (LP) (*n* = 13 in each group; **p < .01; ***p < .001, dots represent observed values).

We did not find any significant difference between the estimates of the total amount of sperm produced by *L. fluviatilis* and *L. planeri* males (LF: 890,392,697 ± 100,990,805 (mean ± SE); LP: 779,163,546 ± 75,045,659; *W* = 94, *p* = .4059). However, *L. planeri* males had a significantly higher sperm concentration than *L. fluviatilis* males (*W* = 15, *p* < .001, Figure 1). Conversely, the VAP was significantly higher for sperm of *L. fluviatilis* males (*t*_24_ = 3.040, *p* < .01, Figure 1). A similar nonsignificant trend was observed for VCL (LF: 353.2 ± 15.4 μm s^−1^; LP: 321.56 ± 17.2 μm s^−1^; *t*_24_ = 1.266, *p* = .218).

**FIGURE 2:**
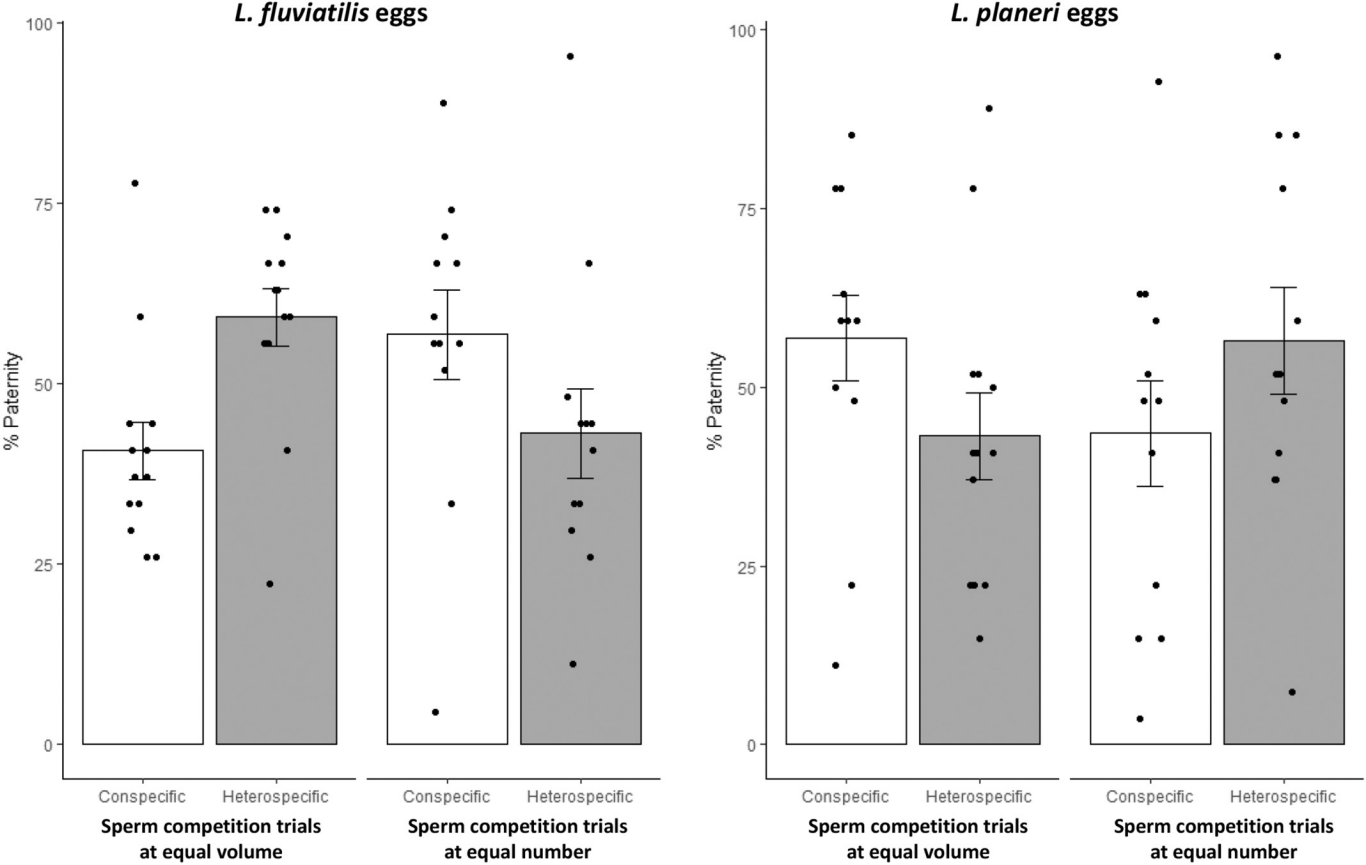
Comparison (mean ± SE) of fertilization success of *Lampetra fluviatilis* and *Lampetra planeri* males in sperm competition trials either at equal semen volume or equal sperm number (dots represent observed values), with either *L. fluviatilis* eggs (left panel) or *L. planeri* eggs (right panel).

**FIGURE 3:**
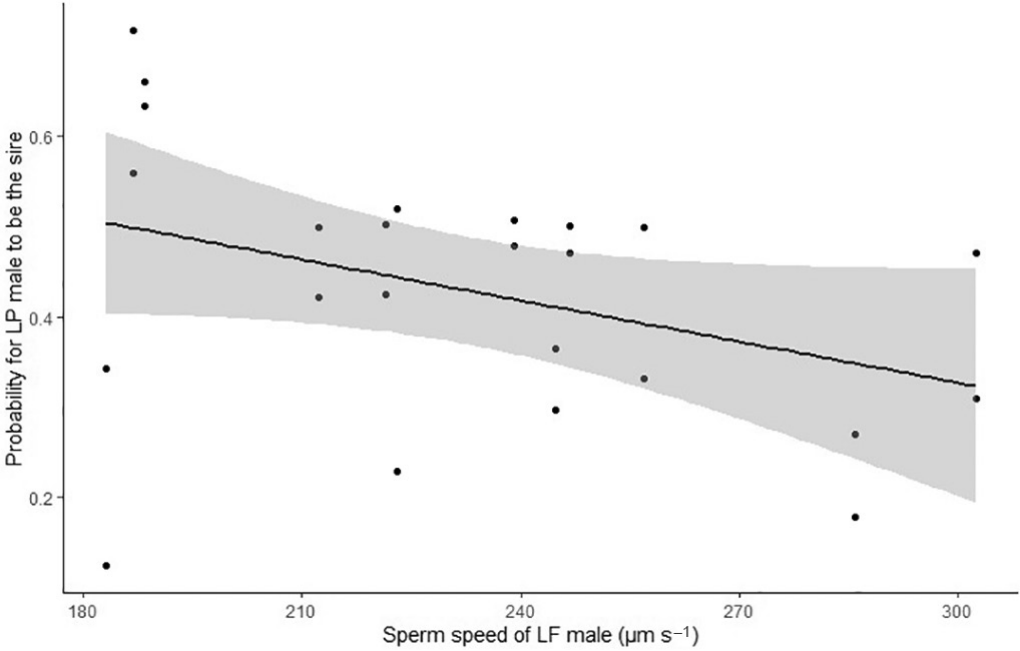
Probability of *Lampetra planeri* (LP) males to sire LP and *Lampetra fluviatilis* (LF) eggs in competitive trials at equal sperm number as a function of LF sperm speed, computed from the Bayesian model. The solid line represents the mean probability, and the gray area gives 95% credible intervals. The dots are observed values.

In noncompetitive fertilization experiments, males of both ecotypes had imilar fertilization success either with *L. planeri* eggs (LF: 0.983 ± 0.008 (mean ± SE); LP: 0.986 ± 0.008; $\chi_{1}^{2}$ = 0.306, *p* = .580) or *L. fluviatilis* eggs (LF: 0.931 ± 0.008 (mean ± SE); LP: 0.949 ± 0.008; $\chi_{1}^{2}$ = 0.360, *p* = .549). In all sperm competition experiments, we found evidence for multipaternity (Figure S1). At equal semen volume, there was a trend for a higher fertilization success of *L. planeri* males: 43.00% ± 0.06 of *L. fluviatilis* progeny (MCMC *p*‐value = .38) and 56.42% ± 0.07 of *L. planeri* progeny (MCMC *p*‐value = .28), with *L. fluviatilis* and *L. planeri* eggs, respectively (Figure 2). By contrast, at equal sperm number, there was an opposite trend with a higher fertilization success of *L. fluviatilis* males: 59.31 ± 0.06% (mean ± SE) of *L. fluviatilis* progeny with *L. fluviatilis* eggs (MCMC *p*‐value = .65) and 43.23 ± 0.06% of *L. planeri* progeny with *L. planeri* eggs (MCMC *p*‐value = .54). However, there was a significant effect of the treatment (equal volume versus equal number, MCMC *p*‐value = 0, Table 1, Figure 2), meaning that the lower fertilization success of LP males at equal sperm number was related to their lower sperm velocity. Accordingly, we found a negative effect of *L. fluviatilis* sperm velocity on the fertilization success of *L. planeri* males in the equal sperm number treatment (MCMC *p*‐value = .046; Figure 3). Finally, no effect of the female ecotype was found on the male fertilization success (Table 1).

**TABLE 1:** Results of GLMM fitted with the Bayesian approach and the proportion of offspring sired by an LP male as the response variable.

Predictor	Mean estimate (±SD)	$\hat{R}$	MCMC *p*‐value
Female ecotype	−0.039 ± 0.684	1.009	1
Experiment type	−0.698 ± 0.120	1.000	<.001
LP sperm speed	−0.184 ± 0.363	1.001	.584
LF sperm speed	0.126 ± 0.410	1.001	1
LP sperm speed * experiment type	0.213 ± 0.250	1.000	.535^(a)^
LF sperm speed * experiment type	−0.833 ± 0.245	1.000	.046^(a)^

*Note*: The experiment type effect designates the sperm competition trials performed either at equal semen volume or equal sperm number. The $\hat{R}$ value is the Gelman‐Rubin statistic that indicates convergence for the estimated parameter when it is <1.1. The MCMC *p*‐values correspond to two‐tailed *t*‐tests except in two cases where one‐tailed tests were computed (a).

## 4 DISCUSSION

This study revealed marked differences between sperm traits of *L. fluviatilis* and *L. planeri*. We found that *L. planeri* males had a sperm almost twice as much concentrated as *L. fluviatilis* males. This result is consistent with the fact that *L. planeri* males have a higher GSI and have been observed to adopt a sneaking tactic (Hume et al., 2013b). “Sneaker” males are usually individuals of small body size who cannot compete with their larger dominant conspecifics (Simmons et al., 1999). The theoretical expectation is that sneaker males should produce more sperm that are more competitive because they are exposed to higher risks of competition than dominant males (Kustra & Alonzo, 2020). Accordingly, some empirical studies indicate that sneaker males tend to produce more sperm (Miller et al., 2019; Poli et al., 2018; Rasotto & Mazzoldi, 2002). We can hypothesize that the smaller body size of *L. planeri* may have led to a strategic sperm allocation with greater sperm number and GSI than *L. fluviatilis* (Wedell et al., 2002). However, we found no difference in the total amount of sperm produced between ecotypes, hence the higher investment in sperm production by *L. planeri* males may “just” compensate for their lower body size, when they try to mate with *L. fluviatilis* females. Nevertheless, the total amount of sperm produced by a male is difficult to precisely estimate as males can regenerate sperm over the spawning season. Importantly, the number of sperm released during mating is not known and may vary between ecotypes.

In addition, *L. planeri* males had a lower sperm velocity than *L. fluviatilis* males, which is opposite to the expected pattern given their sneaking strategy. One might hypothesize that higher sperm velocity in *L. fluviatilis* may be an adaptation to spawning under higher water flow conditions as *L. planeri* spawning sites are usually found in areas with a lower water velocity (i.e., headwater streams) than those of *L. fluviatilis*. Alternatively, there might be a trade‐off between sperm concentration and sperm quality, as *L. planeri* males may strongly invest in sperm numbers at the cost of a lower sperm quality (Snook, 2005). A trade‐off may also occur between sperm velocity and sperm morphology as observed in ocellated wrasses where sneaker males have sperm cells with larger heads than satellite or nesting males and their sperm are also slower (Alonzo et al., 2021).

We performed between‐ecotype crosses to confirm the absence of any genetic incompatibility at least until the developmental stage we used to measure the siring success in sperm competition trials. Accordingly, in the absence of sperm choice (i.e., with sperm of a single male), no barrier to hybridization between sperm and ova in *L. fluviatilis* and *L. planeri* was found. This result is consistent with other hybridization experiments between these taxa (Hume et al., 2013a; Rougemont et al., 2015; Staponkus & Kesminas, 2014). In sperm competition experiments, females did not favor the sperm of males from the same ecotype, so we found no evidence for cryptic female choice. We can thus assume that sexual selection at the postmating prezygotic level between *L. fluviatilis* and *L. planeri* level is primarily driven by sperm traits. However, it is important to remind that ecotypes studied here were collected in a sympatric population. We could hypothesize that in allopatric populations in absence of *L. fluviatilis*, the sperm number of *L. planeri* males could be lower with no evolution of a sneaking strategy in those populations. Further studies could thus compare sperm performances between *L. planeri* populations in allopatry and sympatry with *L. fluviatilis*.

The competitive trials confirmed that sperm concentration and velocity can be considered as predictors of paternity during sperm competition as *L. planeri* males sired significantly less progeny in trials at equal sperm numbers compared with those at equal semen volume. In addition, significant effects of the sperm velocity of *L. fluviatilis* males on fertilization success were highlighted with *L. planeri* eggs for both types of sperm competitions. These results are consistent with other studies in fish with external fertilization, which demonstrated that fertilization success reflects sperm velocity when the effect of sperm number is suppressed (Gage et al., 2002, 2004).

Other sperm traits could also influence the outcome of sperm competition. For instance, a trade‐off between sperm velocity and longevity (i.e., the duration of forward motility, Turner & Montgomerie, 2002) might occur as predicted by sperm competition theory (Parker & Pizzari, 2010). Sperm longevity could influence fertilization success notably when gamete release by males and females is not synchronous (Staponkus & Kesminas, 2014). In addition, the morphology of sperm could also be compared between *L. fluviatilis* and *L. planeri* as it might influence sperm velocity and siring success (Alonzo et al., 2021).

To conclude, our results indicate that at equal semen volume, *L. planeri* males had a higher fertilization success than *L. fluviatilis* males due to higher sperm concentration. By contrast, at equal sperm number, *L. fluviatilis* males sired more offspring due to their higher sperm velocity. These results are similar irrespective of female ecotype and thus show an absence of cryptic female choice. In absence of any postmating prezygotic barrier, our results further support the hypothesis of two partially reproductively isolated ecotypes. As a result, sperm traits are unlikely a determining factor in the speciation process, and the main reproductive barrier in this system might be the size of assortative mating, although it has not been precisely quantified (Beamish & Neville, 1992; Malmqvist, 1983). In addition, genomic incompatibilities (i.e., postzygotic barriers) beyond the larval stage could also contribute to reproductive isolation if hybrids have a lower survival or reduced sperm performances (Whiteley et al., 2009).

## AUTHOR CONTRIBUTIONS

**Nolwenn Decanter:** Formal analysis (lead); writing – original draft (equal); writing – review and editing (equal). **Romane Normand:** Formal analysis (equal); writing – original draft (lead). **Ahmed Souissi:** Methodology (equal). **Catherine Labbe:** Methodology (equal). **Eric Edeline:** Formal analysis (equal). **Guillaume Evanno:** Conceptualization (lead); writing – review and editing (equal).

## FUNDING INFORMATION

This research received no specific grant from any funding agency in the public, commercial, or not‐for‐profit sectors.

## CONFLICT OF INTEREST STATEMENT

All authors declare that they have no conflicts of interest.

## Supporting information


Appendix S1

## Data Availability

The data that support the findings of this study are openly available in Dryad at https://doi.org/10.5061/dryad.5qfttdzb1
