# Supplementary material for: Sperm competition experiments reveal low prezygotic postmating isolation between parasitic and nonparasitic lamprey ecotypes
Source: Ecol Evol. 2023 Apr 2;13(4):e9970. doi: 10.1002/ece3.9970 (PMC10067809; doi:10.1002/ece3.9970)
Supplement: Supplementary file 1 — Appendix S1 [file ECE3-13-e9970-s001.docx]

Supplementary materials

**Table S1**. Settings used in OpenCASA to assess the sperm velocity parameters.

| Microns per Pixel | 0.45 |
| --- | --- |
| Minimum cell size (um^2) | 20.0 |
| Maximum cell size (um^2) | 200.0 |
| Frame Rate (frames/s) | 60.0 |
| Minimum Track Length (frames) | 10 |
| Maximum displacement between frames (um) | 20.0 |
| Window Size (frames) | 4 |

**Table S2**. Females and male pairs combinations used in this experiment on either the 22^nd^, 23^rd^, 29^th^ or 30^th^ of April 2021. Each male pair was used in sperm competition trials with two females at the date indicated in the table. Each male was also used for non-competitive fertilization trials with two females, at the same date it was used for sperm competition trials. All individuals were only stripped once on a single day, except female LF A that was stripped twice on April 22^nd^ and 23^rd^. LP = *Lampetra planeri*; LF = *Lampetra fluviatilis*.

|  | **Female** | | | | | | | | |
| --- | --- | --- | --- | --- | --- | --- | --- | --- | --- |
| **Male pair** | LF A | LP A | LP B | LF B | LF C | LP C | LP D | LP E | LP F |
| LP 1 + LF 1 | 22 | 22 |  |  |  |  |  |  |  |
| LP 2 + LF 2 | 22 | 22 |  |  |  |  |  |  |  |
| LP 3 + LF 3 | 22 | 22 |  |  |  |  |  |  |  |
| LP 4 + LF 4 | 22 | 22 |  |  |  |  |  |  |  |
| LP 5 + LF 5 | 23 |  | 23 |  |  |  |  |  |  |
| LP 6 + LF 6 | 23 |  | 23 |  |  |  |  |  |  |
| LP 7 + LF 7 | 23 |  | 23 |  |  |  |  |  |  |
| LP 8 + LF 8 |  |  |  | 29 |  | 29 |  |  |  |
| LP 9 + LF 9 |  |  |  | 29 |  | 29 |  |  |  |
| LP 10 + LF 10 |  |  |  | 29 |  |  | 29 |  |  |
| LP 11 + LF 11 |  |  |  | 29 |  |  | 29 |  |  |
| LP 12 + LF 12 |  |  |  | 29 |  |  |  | 29 |  |
| LP 13 + LF 13 |  |  |  |  | 30 |  |  |  | 30 |


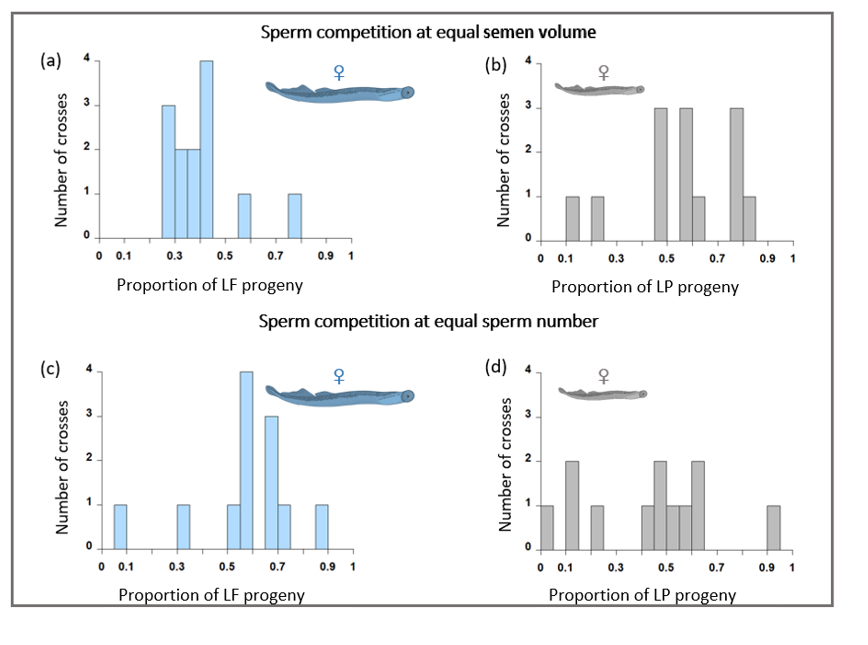


**Figure S1.** Representation of the outcomes of sperm competition experiments at equal semen volume and at equal sperm number. In (a) and (c), we represented the proportion of *L. fluviatilis* (LF) eggs that were fertilized by *L. fluviatilis* males (blue). In (b) and (d), we represented the proportion of *L. planeri* (LP) eggs that were fertilized by *L. planeri* males (grey).


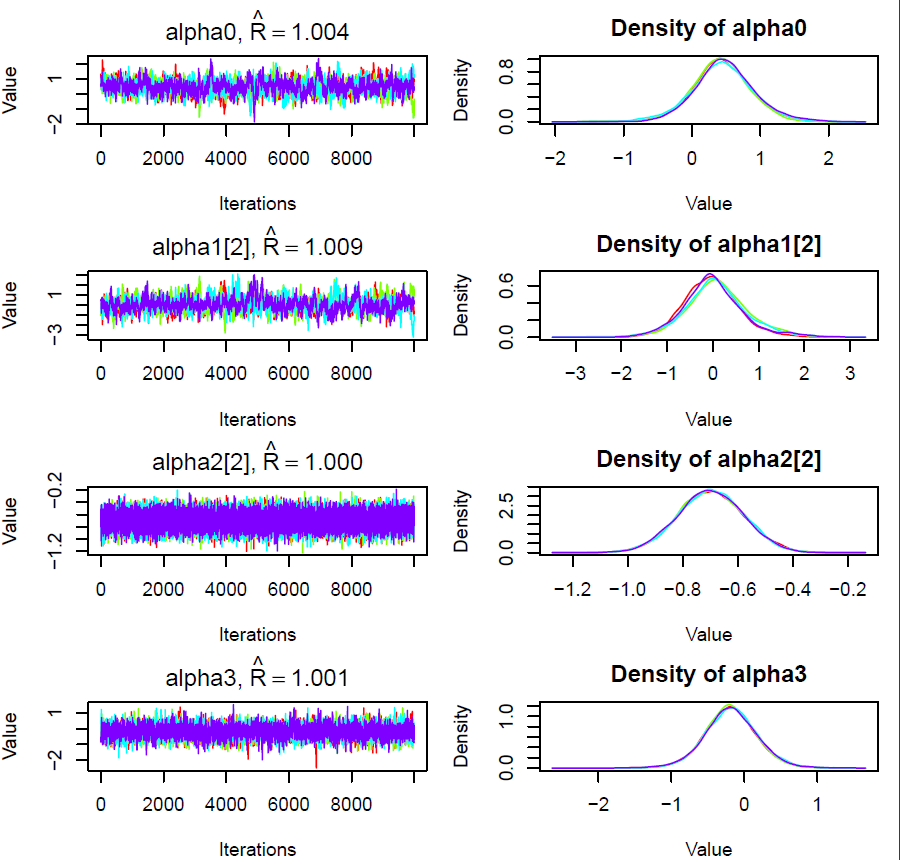

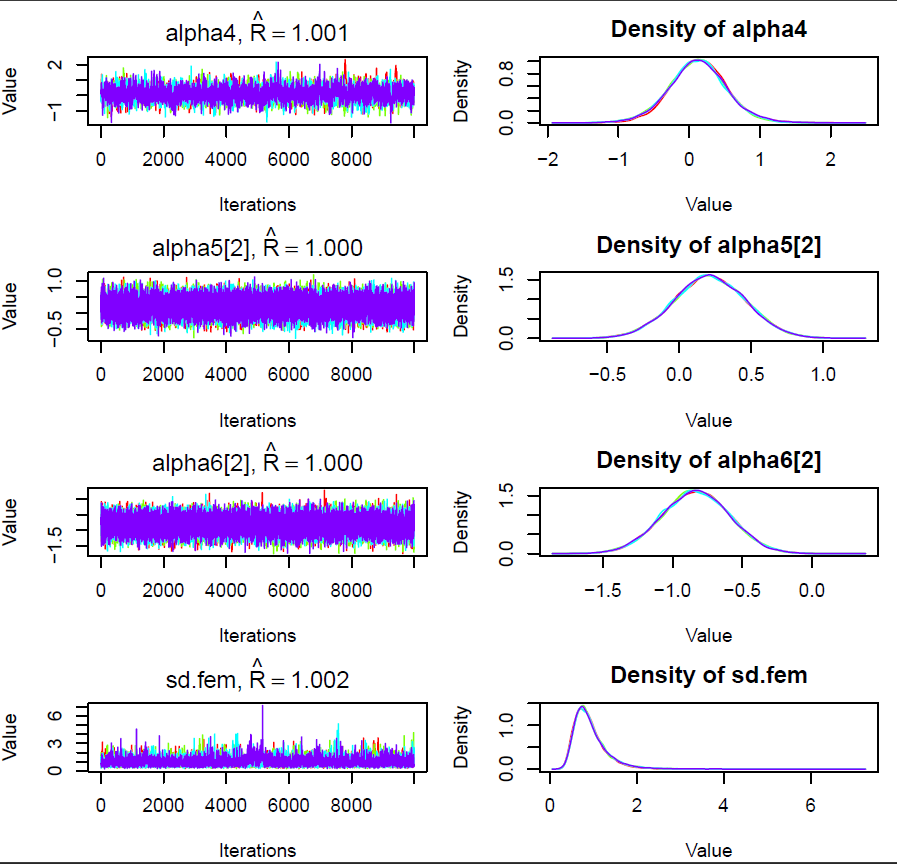

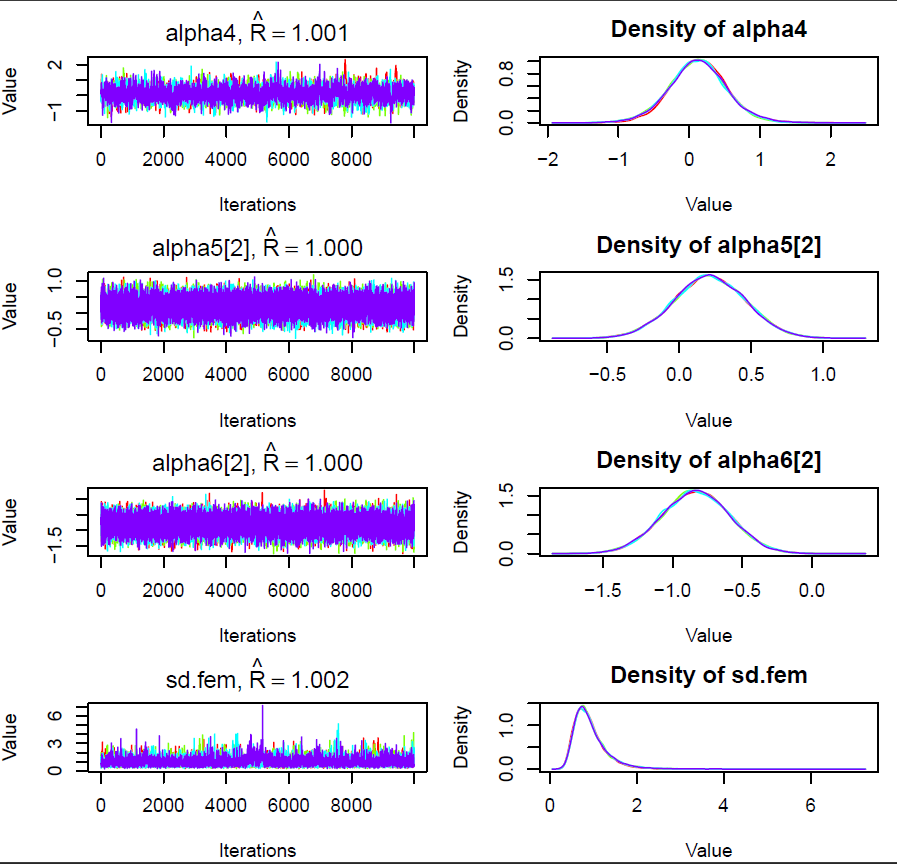


**6))**

**5)**

**4)**

**3)**

**1)**

**2)**

**7))**


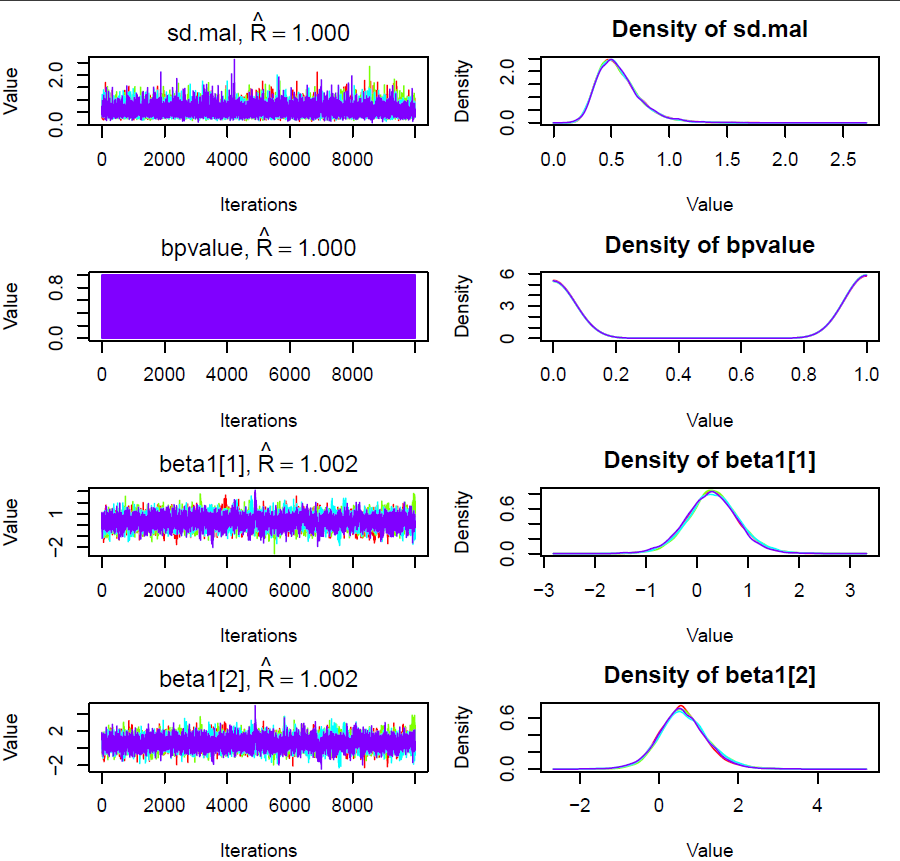

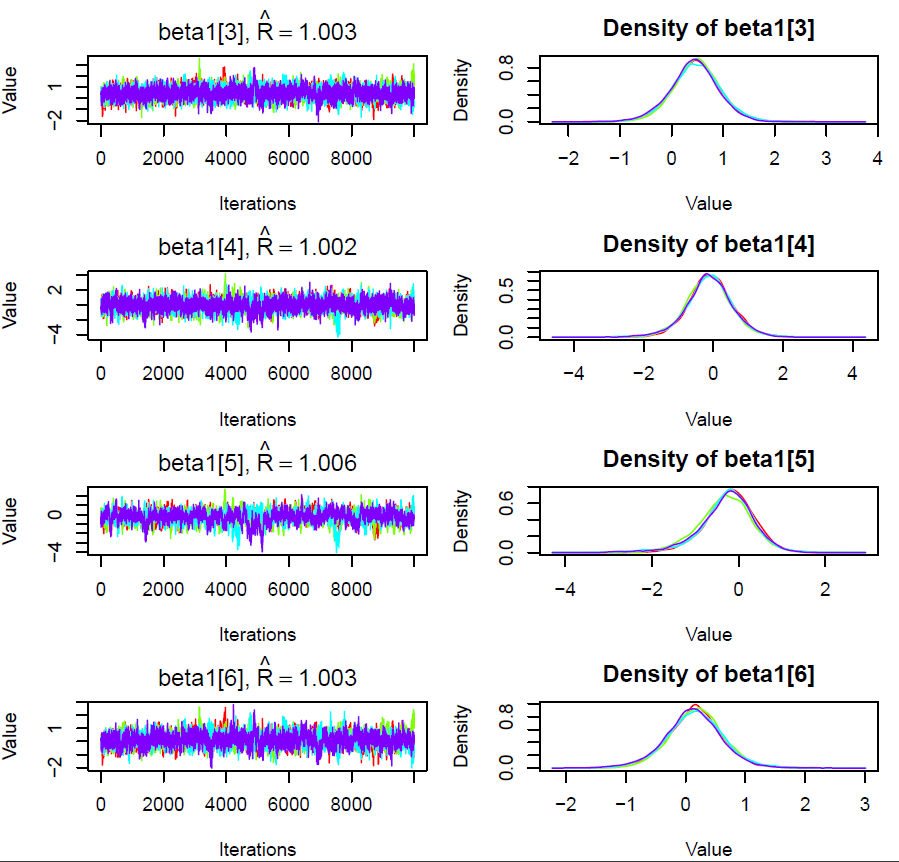

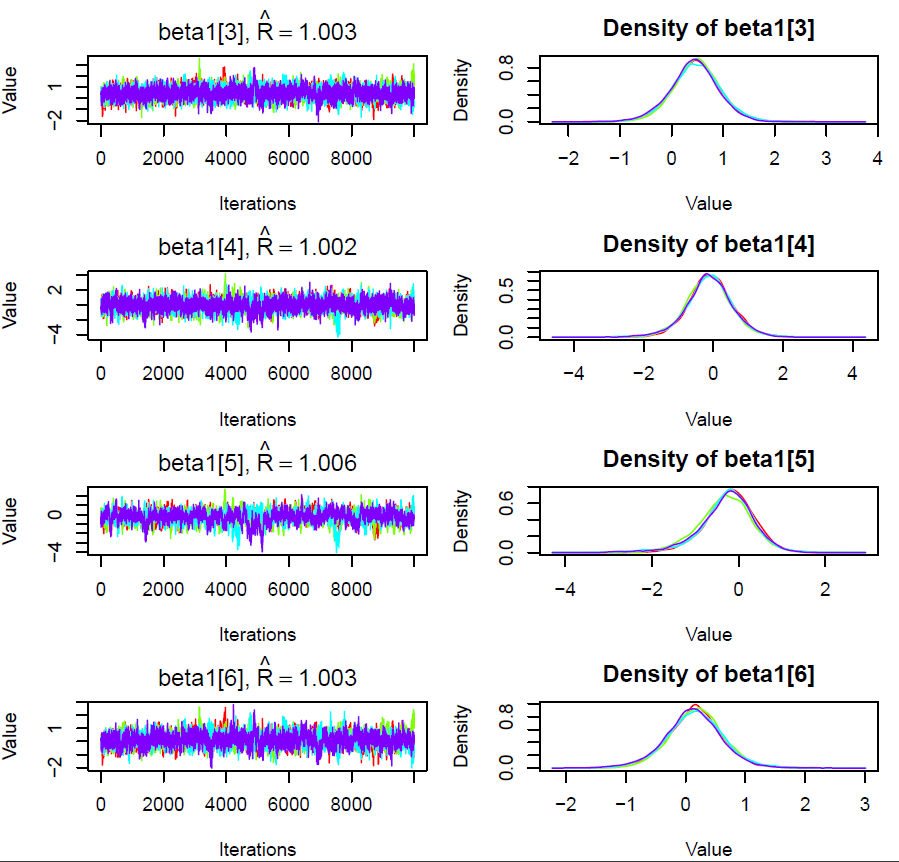

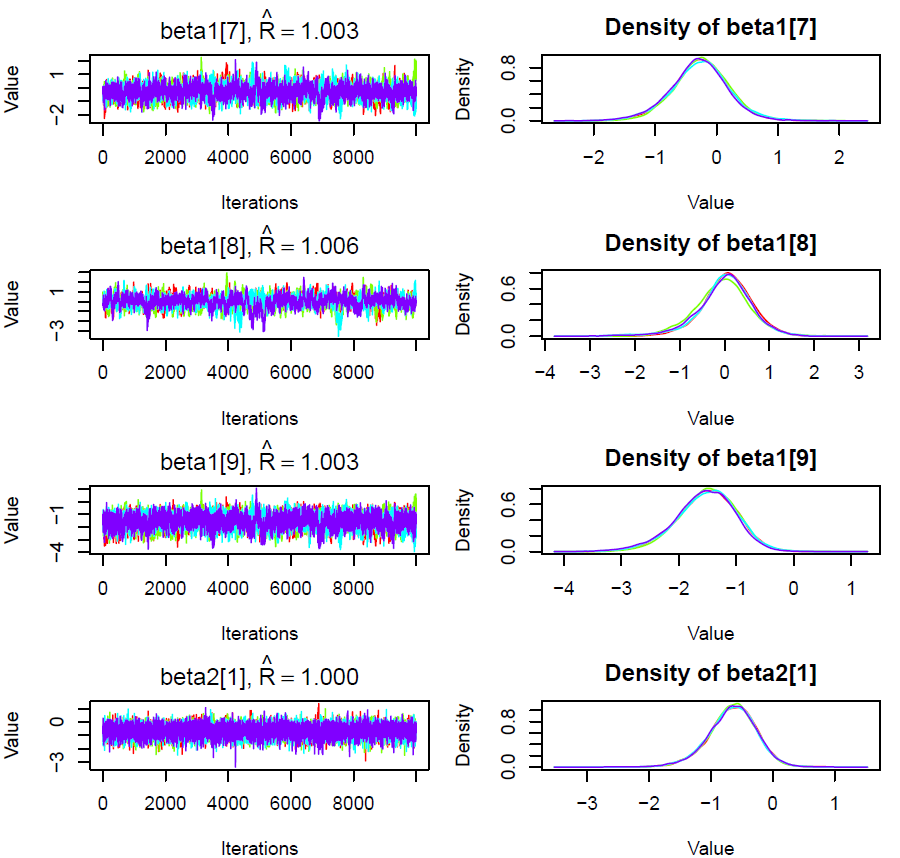


**12))**

**11))**

**10))**

**9))**

**8))**

**13))**

**15))**

**14))**


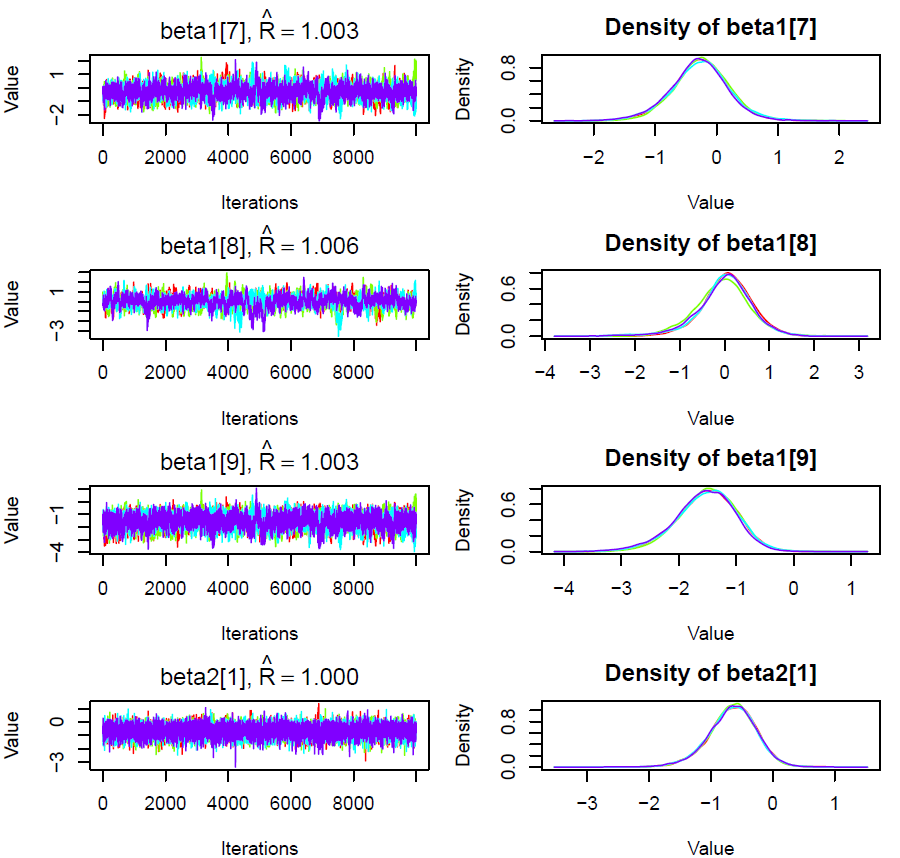


**17))**

**16))**


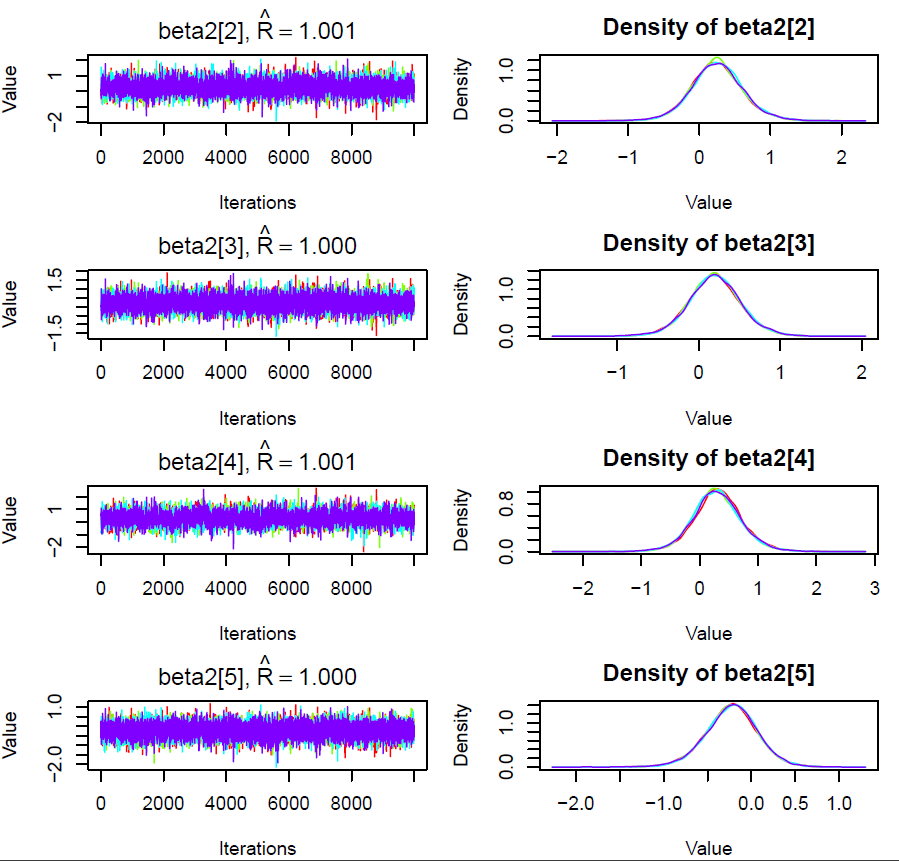

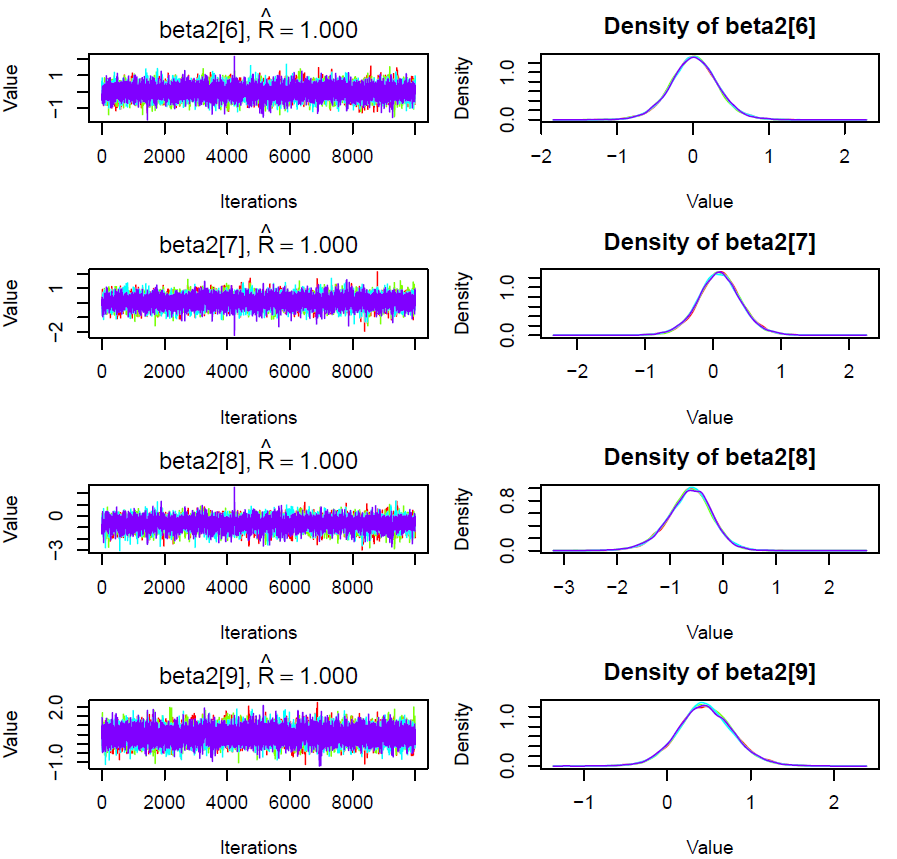

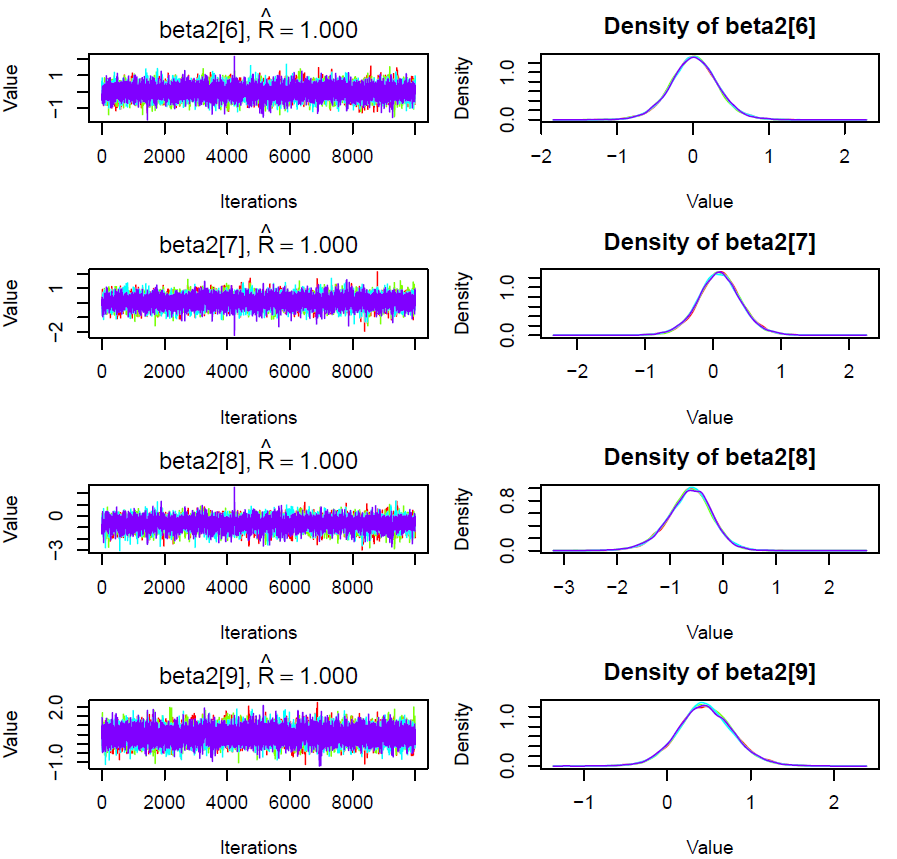


**23))**

**22))**

**21))**

**20))**

**19))**

**18))**

**25))**

**24))**


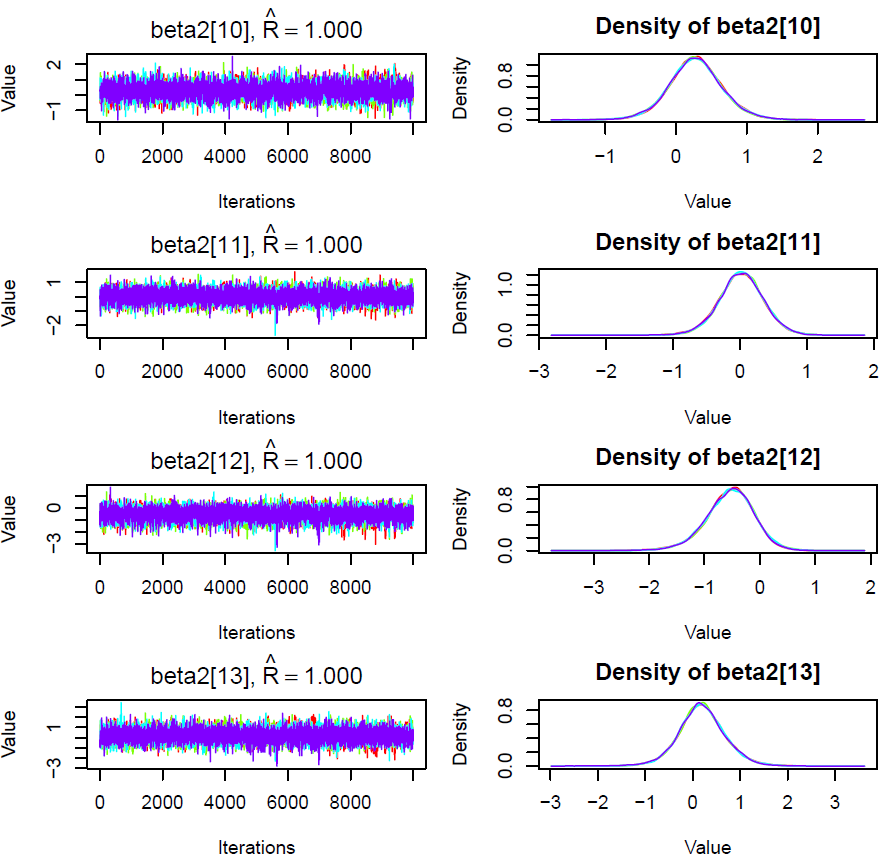


**29)**

**28))**

**27))**

**26))**

**Figure S2. Trace plots and density plots of the Bayesian model.** 1) to 7) correspond to the fixed effects of the model. $\propto_{0}$ is the fixed effect intercept; $\propto_{k^{'s}}$, $k\in\{1,\ldots,6\}$ are the fixed effects associated with the female ecotype (LP or LF), the experiment type, the sperm speed of LP, the sperm speed of LF, the interaction between the sperm speed of LP and the experiment type, and the interaction between the sperm speed of LF and the type of experiment, respectively. From 8) to 17) are the plots of the random effect associated with the female identity and from 18) to 29) are the plots associated to $\beta_{2},$ the male pair random effect.

**Table S3:** Estimates of random effects in GLMMs fitted with the Bayesian approach. Female IDs are denoted β_1[i]_ and male pairs are noticed β_2[i]_ .

| Random effect parameter | Mean estimate (± SD) | R-hat |
| --- | --- | --- |
| $\beta_{1[1]}$ | 0.314 ± 0.522 | 1,002 |
| $\beta_{1[2]}$ | 0.619 ± 0.626 | 1,002 |
| $\beta_{1[3]}$ | 0.472 ± 0.484 | 1,003 |
| $\beta_{1[4]}$ | -0.106 ± 0.677 | 1,002 |
| $\beta_{1[5]}$ | -0.261± 0.631 | 1,006 |
| $\beta_{1[6]}$ | 0.160 ± 0.477 | 1,003 |
| $\beta_{1[7]}$ | -0.263 ± 0.476 | 1,003 |
| $\beta_{1[8]}$ | 0.007 ± 0.615 | 1,006 |
| $\beta_{1[9]}$ | -1.524 ± 0.530 | 1,003 |
| $\beta_{2[1]}$ | -0.651 ± 0.390 | 1,000 |
| $\beta_{2[2]}$ | 0.253 ± 0.369 | 1,001 |
| $\beta_{2[3]}$ | 0.198 ± 0.326 | 1,000 |
| $\beta_{2[4]}$ | 0.301 ± 0.422 | 1,001 |
| $\beta_{2[5]}$ | -0.225± 0.300 | 1,000 |
| $\beta_{2[6]}$ | 0.013 ± 0.320 | 1,000 |
| $\beta_{2[7]}$ | 0.093 ± 0.326 | 1,000 |
| $\beta_{2[8]}$ | -0.635± 0.436 | 1,000 |
| $\beta_{2[9]}$ | 0.463 ± 0.340 | 1,000 |
| $\beta_{2[10]}$ | 0.279 ± 0.382 | 1,000 |
| $\beta_{2[11]}$ | 0.023 ± 0.341 | 1,000 |
| $\beta_{2[12]}$ | -0.542± 0.444 | 1,000 |
| $\beta_{2[13]}$ | 0.199 ± 0.497 | 1,000 |
